# EGFR Exon 18 Mutations in East Asian Patients with Lung Adenocarcinomas: A Comprehensive Investigation of Prevalence, Clinicopathologic Characteristics and Prognosis

**DOI:** 10.1038/srep13959

**Published:** 2015-09-10

**Authors:** Chao Cheng, Rui Wang, Yuan Li, Yunjian Pan, Yang Zhang, Hang Li, Difan Zheng, Shanbo Zheng, Xuxia Shen, Yihua Sun, Haiquan Chen

**Affiliations:** 1 Department of Thoracic Surgery, Fudan University Shanghai Cancer Center, Shanghai, 200032, China; 2 Department of Oncology, Shanghai Medical College, Fudan University, Shanghai, 200032, China; 3 Department of Pathology, Fudan University Shanghai Cancer Center, Shanghai, 200032, China; 4 Shanghai Chest Hospital, Shanghai Jiao Tong University, Shanghai, 200030, China; 5 Institutes of Biomedical Sciences, Fudan University, 130 Dong-An Road, Shanghai, 200032, China

## Abstract

Our aim was to investigate the clinical and pathologic characteristics of the epidermal growth factor receptor (*EGFR*) exon 18 mutations in East Asian lung adenocarcinomas patients. A total of 1,201 lung adenocarcinomas were analyzed for mutation in *EGFR*. Clinical and pathologic characteristics of patients with *EGFR* exon 18 mutations were compared with those who harbored classic activating mutations (exon 19 deletions and the L858R point mutation). The mutations in *EGFR* exon 18 were observed in 2.8% of 1,201 lung adenocarcinomas and 4.6% of patients with *EGFR* mutations. Patients with a single *EGFR* exon of 18 mutations had a worse overall survival than those harboring the complex *EGFR* exon of 18 mutations (*p* = 0.002) or those with classic activating mutations (*p* = 0.014). Four of five patients with *EGFR* exon 18 mutations showed objective response to the EGFR-TKI therapies after disease recurrence. Our results demonstrated that single *EGFR* exon 18 mutations may be an indicator of poor prognosis compared with complex *EGFR* exon 18 mutations or classic mutations. Furthermore, the results of the current study will be helpful for decision-making in the treatment of patients with *EGFR* exon 18 mutations.

Lung cancer is the leading cause of cancer-related death worldwide, and non–small cell lung cancer (NSCLC) is the most common type of this disease^1^,^2^. With comprehensive understanding of the genetic alteration of lung cancer, many onco-targeted drugs had been developed, and great achievements have been attained in patients with advanced disease^3^,^4^,^5^,^6^,^7^,^8^.

Patients with activating *EGFR* mutations are identified in ~20% of lung adenocarcinomas in Western countries^9^ and 40~60% of lung adenocarcinomas in East Asia^10^,^11^,^12^. These mutations mainly consist of in-frame deletions in exon 19 (~50%) and the L858R point mutation in exon 21 (~40%)^9^, and they are associated with a favorable response to the EGFR tyrosine kinase inhibitors (EGFR-TKI), such as gefitinib and erlotinib^5^,^13^. Exon 19 and 21 are located in the intracellular kinase domain of *EGFR*, which also includes exons 18 and 20^4^.

Both point mutations in exon 18 and insertion mutations in exon 20 are relatively infrequent, respectively, at 3% and 5% of the *EGFR* mutations^14^,^15^. With more than 1.6 million cases of lung cancer diagnosed and 1.3 million deaths per year^16^, even small subgroups of NSCLC contribute to significant morbidity and mortality. Insertions in exon 20 of *EGFR* have been reported to be associated with resistance to EGFR-TKI and poor prognosis in NSCLC patients^17^,^18^,^19^,^20^. Single point mutations in exon 18 mainly consist of E709X and G719X mutations. Those mutations have been identified in several previous studies with limited sample sizes^11^,^21^,^22^,^23^. However, other complex *EGFR* mutations that included not only single point mutations in exon 18 but also other genetic alterations in the EGFR kinase domain were not well characterized. In addition, the relationship between the complex mutations and sensitivity to EGFR-TKI therapy has not been completely elucidated.

In this study, we retrospectively investigated the frequency, molecular spectrum and clinicopathologic characteristics of patients with *EGFR* exon 18 mutations in a large cohort of patients with lung adenocarcinomas. We also analyzed lung cancer patients with single or complex *EGFR* exon 18 mutations and their correlation to treatment outcome with EGFR-TKI.

## Results

A total of 1,201 patients with lung adenocarcinomas were screened for *EGFR* mutation status. Of those, 737 (61.4%) patients were found to harbor mutations in *EGFR*. Among the patients who harbored *EGFR* mutations, we detected 34 (4.6% of 737) patients with mutations in the *EGFR* exon 18,661 (89.7%) cases with classic activating mutations (exon 19 deletions and L858R point mutation), and 42 patients harbored other rare mutations.

Of the 34 patients with *EGFR* exon 18 mutations, 27 (79.4%) were women, and 29 (85.3%) were never-smokers. The amino acid sequence of the *EGFR* exon 18 mutations included 23 different variants, and only 4 of 23 variants occurred more than once. Seventeen (50.0%) variants involved the G719 locus, 7 (20.6%) variants involved the E709 locus and 4 (11.8%) involved both G719 and E709, with 6 (17.6%) others. The predominant pathological subtype included 16 (47.1%) with acinar tumors, 8 (23.5%) with papillary tumors, 4 (11.8%) with solid tumors, 4 (11.8%) with lepidic tumors and 2 (5.9%) with minimally invasive adenocarcinoma (MIA) (Table 1).

The clinicopathologic characteristics for each individual patient who carried mutations in the *EGFR* exon 18 are shown in Table 1. No significant differences were identified between the patients carrying a mutation in exon 18 and those with activating mutations regarding age, smoking status, stage, tumor size and differentiation (Table 2).

**Figure 1:**
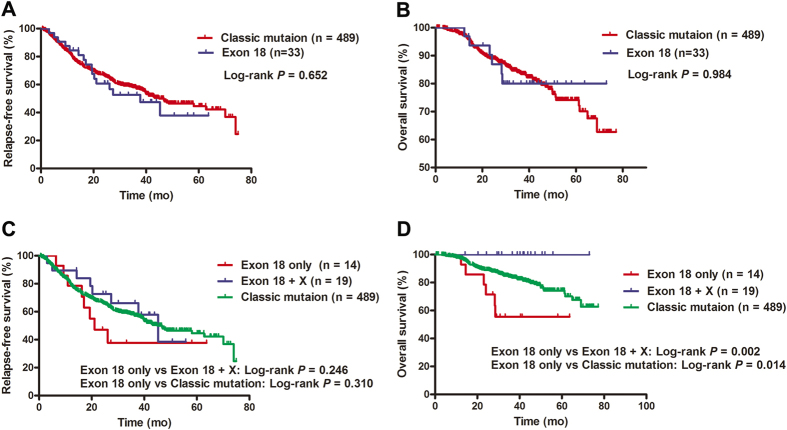
Recurrence-free survival (RFS) and overall survival (OS) of cancers with *EGFR* exon 18 mutations. Kaplan–Meier survival curves for RFS (**A**) and OS (**B**) analyses between classic activating mutations and exon 18 mutations. Kaplan–Meier survival curves for RFS (**C**) and OS (**D**) analyses among single exon 18 mutations (indicated as ‘exon 18 only’), complex exon 18 mutations (indicated as ‘exon 18 + X’) and classic mutations. The log-rank test is used.

A total of 33 patients with *EGFR* exon 18 mutations and 489 patients with classic activating mutations diagnosed from October 2007 to March 2011 were included for survival analysis. The median follow-up duration of these patients was 33 months (range: 1–78 months). There were no significant differences in RFS (*p* = 0.652, Fig. 1A) and OS (*p* = 0.984, Fig. 1B) between patients with *EGFR* exon 18 mutations and patients with classic activating mutations. We further divided patients with exon 18 mutations into two subgroups: patients with single *EGFR* exon 18 mutations and those with complex *EGFR* exon 18 mutations (*EGFR* exon 18 mutations + other *EGFR* mutations). In univariate analysis, although there were no significant differences in RFS between those two subgroups (log-rank *p* = 0.246, Fig. 1C) and between single *EGFR* exon 18 mutations and classic mutations (log-rank *p* = 0.310, Fig. 1C), the OS of patients with single *EGFR* exon 18 mutations was much worse than those with complex *EGFR* exon 18 mutations (log-rank *p* = 0.002, Fig. 1D) or those with classic mutations (log-rank *p* = 0.014, Fig. 1D). Gender (*p* = 0.003), smoking history (*p* < 0.001), tumor size (*p* < 0.001), stage (*p* < 0.001) and differentiation (*p* < 0.001) were significantly associated with RFS. Smoking history (*p* = 0.017), tumor size (*p* < 0.001), stage (*p* < 0.001) and differentiation (*p* < 0.001) were significantly correlated with a worse OS (Supplementary Table S1). In multivariate analysis incorporating mutation status, gender, age, smoking history, tumor size (≤3 vs. >3 cm), stage (I vs. II–IV), differentiation (well/moderate vs. poor), smoking history (hazard ratio = 2.184, 95% confidence interval: 1.393–3.422, *p* = 0.001), tumor size (hazard ratio = 1.397, 95% confidence interval: 1.057–1.845, *p* = 0.019), stage (hazard ratio = 4.763, 95% confidence interval: 3.485–6.510, *p* < 0.001) and differentiation (hazard ratio = 1.608, 95% confidence interval: 1.199–2.156, *p* = 0.002) were the independent predictor of RFS, and single exon 18 mutations (hazard ratio = 2.239, 95% confidence interval: 1.005–4.989, *p* = 0.049), tumor size (hazard ratio = 1.917, 95% confidence interval: 1.247–2.947, *p* = 0.003), stage (hazard ratio = 5.644, 95% confidence interval: 3.064–10.396, *p* < 0.001) and differentiation (hazard ratio = 2.036, 95% confidence interval: 1.310–3.165, *p* = 0.002) were the independent predictor of OS (Supplementary Table S2).

Nine patients received platinum-based combination chemotherapies, and one patient received fluorouracil. Of these, two patients received neoadjuvant chemotherapies before surgery, and eight received chemotherapies after disease recurrence. Of the two patients who received neoadjuvant chemotherapies, according to Response Evaluation Criteria in Solid Tumors (RECIST), one had a RECIST stable disease and the other had a RECIST partial response. Of the eight patients receiving chemotherapy, one had a RECIST partial response, two had RECIST stable disease, four had RECIST progressive disease, and one was of unknown status because of loss to follow-up (Table 1). The only one patient receiving radiotherapy after disease recurred had a RECIST stable disease (Table 1).

After the disease relapsed, five patients with *EGFR* exon 18 mutations received EGFR-TKI therapies, including 2 treated with erlotinib and 3 with gifitinib as first-, second- or third-line therapy. According to RECIST, four patients had a RECIST partial response. The times to progression for these patients were 65.0, 14.8+ 20.7+ and 37.5+ months, respectively, and the overall survival after taking the TKIs were 68+, 14.8+, 29.4+, and 37.5+ months, respectively (Table 3). One patient had a RECIST stable disease, and both the RFS and OS were 24.2+ months (Table 3).

## Discussion

The management of lung adenocarcinomas has been transformed by the identification of targetable oncogenic drivers that confer sensitivity to specific tyrosine kinase inhibitors. Activating mutations in the *EGFR* gene, such as a deletion on exon 19 and a point mutation in exon 21, identifies a distinct subset of lung cancers that are uniquely sensitive to EGFR-TKIs. In the current study, we demonstrated that exon 18 mutations represent an additional target that is sensitive to EGFR-TKI therapy, regardless of less common than classic *EGFR* mutations.

In the current study, five patients who harbored *EGFR* exon 18 mutations had a favorable RFS after receiving the TKI therapies. The response rates (4/5) and RFS of our cohort were greater than those reported by other groups^21^,^22^,^24^,^25^,^26^. This may be due to the ethnicity and small sample size of our cohort. In addition, one patient harbored double point mutations (G719S+L861Q) and obtained an amazing RFS of 65 months, which causes us to explore the potential effect of TKI therapies on different *EGFR* mutations. We also found that two patients benefited from TKI therapies after failure to ameliorate the tumor progression by chemotherapies. Furthermore, therapy for one patient failed to ameliorate the tumor progression by radiotherapy and to obtain similar improvement. Taken together, our results suggest that TKI therapies should be considered as a prior choice for advanced lung adenocarcinoma patients with *EGFR* mutations in exon 18.

Although some previous reports showed the discordance of EGFR mutation between primary and metastatic tumors^27^,^28^, further studies with large sample sizes and studies utilizing the high throughput technology of whole exome sequencing demonstrated that driver events, such as EGFR and BRAF mutations, were highly consistent between primary and metastatic tumors. Given that the samples were obtained mainly by aspiration biopsy and FFPE tissues^29^,^30^, the quality and quantity might not be enough to obtain an accurate EGFR mutation status.

Survival analysis results showed that there were no significant differences in RFS and OS between *EGFR* mutations in exon 18 and classic activating mutations. A comparison of the Kaplan-Meier curves suggested that the OS of patients with single exon 18 mutations was shorter than those with complex exon 18 mutations or patients with classic *EGFR* mutations. However, there were no significant differences in RFS between patients with single exon 18 mutations and those with complex exon 18 mutations or classic mutations. Our results indicate that single *EGFR* exon 18 mutations may be an indicator of poor prognosis compared with classic activating mutations or complex exon 18 mutations. Further investigations are required to address these differences.

To our knowledge, this report is the first comprehensive study of clinicopathologic features of *EGFR* exon 18 mutations in a large cohort of patients with lung adenocarcinoma. We showed that *EGFR* mutations in exon 18 were present in 2.8% of lung adenocarcinomas and 4.6% of *EGFR* mutations, which were similar to the prevalence of *EGFR* exon 20 insertion mutations in East Asians as we previously reported^31^. A limited number of cases with *EGFR* exon 18 mutations had been reported by previous studies^22^,^25^. It is therefore difficult to draw conclusions as to their true prevalence, molecular spectrum, and clinicopathologic features. We found 23 kinds of variants of *EGFR* exon 18 mutations in the current study. Of all exon 18 mutations, G719X mutations in *EGFR* exon 18 were the most common variant, and E709X mutations were the second most common, which was similar to a previous report^22^. We also showed that patients harboring *EGFR* exon 18 mutation had clinicopathologic characteristics very similar to those with classic *EGFR* activating mutations, which were characterized as being more frequent in females and never smokers^10^,^32^.

There are several limitations of this study. First, the finding that patients with single exon 18 mutations had a significantly worse OS than those with complex exon 18 mutations was based on a small number of patients, which needs to be validated in a larger series of patients. Second, we conducted cDNA-PCR sequencing as the major experimental method to identify mutations. Results obtained by analyzing corresponding data involving those from the negative EGFR mutations group might change if more sensitive methods, such as the amplification refractory mutation system (ARMS), are used.

In conclusion, our data demonstrated that *EGFR* exon 18 mutations occurred in 2.8% of patients with NSCLCs and 4.6% of patients with *EGFR* mutations. Single *EGFR* exon 18 mutations may be an indicator of poor prognosis compared with classic activating mutations. Given that 4 of 5 patients with *EGFR* mutations in exon 18 had an objective response to the TKIs therapies and a RFS of 65.0, 14.8+, 20.7+, and 37.5+ months in our cohort, we suggest that advanced patients with those mutations should have TKIs as prior therapy.

## Methods

### Patients and Samples

From October 2007 to January 2013, we consecutively collected lung tumors resected at the Department of Thoracic Surgery, Fudan University Shanghai Cancer Center, Shanghai, China. Inclusion criteria for this study were as follows: (1) patients underwent complete resection with curative intent, and (2) specimens were pathologically confirmed as lung adenocarcinomas with a minimum of 50% of tumor cells and sufficient tissue for comprehensive mutational analyses.

Pathologic slides were reviewed by two certified pathologists (Xuxia Shen and Yuan Li) to classify histologic subtypes of lung adenocarcinomas according to the IASLC/ATS/ERS multidisciplinary classification system^33^. The following clinicopathologic parameters for each patient were also collected: gender, age at diagnosis, smoking history, systemic treatment of advanced lung cancers, and pathologic TNM stage in line with the seventh edition of the lung cancer staging system^34^. Recurrence-free survival (RFS) and overall survival (OS) of patients diagnosed from October 2007 to March 2011 (because of relatively insufficient follow-up duration) were recorded based on a follow-up clinic visit or a telephone call.

### Mutational Analysis

After frozen tumor specimens were dissected in TRIzol reagent (Invitrogen, Carlsbad, CA), DNA and RNA were extracted as per standard protocol, and the RNA was reverse transcribed into cDNA by a RevertAid First Strand cDNA Synthesis Kit (Fermentas, EU). *EGFR* (exons 18–21) were amplified by PCR routinely using cDNA. Direct dideoxynucleotide sequencing was then performed to analyze the amplified products. The *EGFR* (exons 18–21) amplified products obtained by PCR using DNA for sequencing were used to confirm the uncommon EGFR mutations. Primers and PCR condition are listed in the supplementary Table 3.

### Statistical Analysis

Pearson’s χ2 test or Fisher’s exact test was used to investigate the correlations between two categorical variables. The association between one categorical variable and one continuous variable was assessed using the independent sample *t*-test. The RFS and OS distribution was analyzed using the Kaplan–Meier method, and log-rank tests were employed for comparisons of RFS or OS between two categories in univariate analysis. Multivariate survival analysis was conducted using the Cox proportional hazards regression (forward likelihood ratio model) to identify independent prognostic factors. Statistical analyses were performed using SPSS (Statistical Package for the Social Sciences) 16.0 software (SPSS Inc., Chicago, IL). All tests were two-tailed. Statistical significance was set at p < 0.05.

### Ethics Statement

This study was conducted in line with the Helsinki Declaration and approved by the Institutional Review Board of the Fudan University Shanghai Cancer Center. Written informed consent was obtained from each patient to allow their biological samples to be genetically analyzed. The experimental protocol of this study was performed strictly in accordance with the guidelines.

## Additional Information

**How to cite this article**: Cheng, C. *et al.* EGFR Exon 18 Mutations in East Asian Patients with Lung Adenocarcinomas: A Comprehensive Investigation of Prevalence, Clinicopathologic Characteristics and Prognosis. *Sci. Rep.* **5**, 13959; doi: 10.1038/srep13959 (2015).

## Supplementary Material

Supplementary files

## Figures and Tables

**Table 1:** Individual patient characteristics.

Case	Stage	Mutation type	Mutated	Sex/Age	Smoking	Pathological	Neoadjuvant	Adjuvant	First-line treatment	Response
			exons		status	subtype				
1	IV	G719S+L861Q	18, 21	70/F	Never	Acinar	None	Cisplatin + Gemcitabine	Gefitinib	PR
2	Ia	709ET=>D	18	51/F	Never	MIA	None	None	None	N/A
3	IIIa	709ET=>D	18	43/M	Ever	Acinar	None	Cisplatin + Docetaxel	Cisplatin + Gemcitabine	PD
4	Ia	G719A	18	71/F	Never	Lepidic	None	None	None	N/A
5	Ib	F723I+L858R	18, 21	58/F	Never	Acinar	None	None	Fluorouracil	N/A
6	IIIa	V689L+L858R	18, 21	43/F	Never	Papillar	None	Cisplatin + Vinorelbine	Radiotherapy	PD
7	Ib	G719A+I768S	18, 20	77/F	Never	Lepidic	None	None	None	N/A
8	Ib	G719S+del746ELREA	18, 19	60/F	Never	Acinar	None	None	None	N/A
9	IIIa	G719A	18	84/M	Never	Acinar	None	None	None	N/A
10	IIIa	E709K+G719S	18	54/F	Never	Acinar	None	Cisplatin + Gemcitabine	None	N/A
11	IIIb	G719A	18	64/M	Ever	Papillar	None	Cisplatin + Vinorelbine	Cisplatin + Gemcitabine	PD
12	Ib	G719A+L861Q	18, 21	74/F	Never	Acinar	None	None	None	N/A
13	IIIa	L692V+L858R	18, 21	63/F	Never	Acinar	Cisplatin + Gemcitabine	Carboplatin + Gemcitabine	None	PR
14	IIIa	G719C+S768I	18, 20	77/M	Ever	Papillar	None	Cisplatin + Gemcitabine	None	N/A
15	IIIa	G719S+T790M	18, 20	66/F	Never	Solid	None	None	Carboplatin + Gemcitabine	PR
16	Ia	G719A+S768I	18, 20	62/F	Never	Lepidic	None	None	None	N/A
17	Ia	E709K+L858R	18, 21	53/F	Never	Acinar	None	None	None	N/A
18	IV	E709K+G719C	18	46/F	Never	Acinar	None	Cisplatin + Gemcitabine	Cisplatin + Pemetrexed	PD
19	Ia	709ET=>D	18	38/F	Never	Acinar	None	None	None	N/A
20	Ia	Q701L+L858R	18, 21	64/F	Never	Acinar	None	None	None	N/A
21	Ia	G724S+S768I	18, 20	47/F	Never	Papillar	None	Cisplatin + Pemetrexed	Cisplatin + Docetaxel	SD
22	IIIa	709ET=>D	18	47/F	Never	Solid	None	None	Carboplatin + Gemcitabine	PD
23	Ia	E709K+L858R	18, 21	58/F	Never	Papillar	None	None	Gefitinib	PR
24	IV	E709A+G719E	18	67/F	Never	Acinar	None	None	None	N/A
25	Ia	G719S	18	84/F	Never	Papillar	None	None	Cisplatin + Gemcitabine	SD
26	Ia	G719C+S768I	18, 20	57/M	Never	Acinar	None	None	None	N/A
27	IIa	G719S+S768I	18, 20	64/F	Never	Solid	None	Carboplatin + Gemcitabine	None	N/A
28	Ia	E709A+G719S	18	74/F	Never	Papillar	None	None	None	N/A
29	IIa	G719A	18	57/M	Ever	Acinar	None	None	None	N/A
30	Ia	I706T+L861Q	18, 21	59/F	Never	MIA	None	None	None	N/A
31	Ia	G719C+S768I	18, 20	54/M	Ever	Lepidic	None	None	None	N/A
32	Ia	G719C+S768I	18, 20	57/F	Never	Acinar	None	None	None	N/A
33	IIa	G719A	18	61/F	Never	Solid	Cisplatin + Pemetrexed	None	None	SD
34	Ib	E709K+K757R	18, 19,	68/F	Never	Papillar	None	None	None	N/A
		+L858R	21							

F, female; M, male; MIA, minimally invasive adenocarcinoma; PD, progressive disease; PR, partial response; SD, stable disease; N/A, not applicable. Response was evaluated against the neoadjuvant or the first-line treatment.

**Table 2:** Comparison of clinical characteristics between NSCLCs harboring classic activating EGFR mutations and EGFR exon 18 mutations.

Variables	Classic activating	Mutations in	*p*
	mutations	exon 18	
	(n = 661)	(n = 34)	
Gender			
Female	420 (63.5%)	27 (79.4%)	
Male	241 (36.5%)	7 (20.6%)	0.067
Age (y)			
Mean	60.0	60.9	
SD	10.0	11.5	0.652
Smoking history			
Ever	140 (21.2%)	4 (11.8%)	
Never	521 (78.8%)	30 (88.2%)	0.276
Tumor size (cm)			
≤3	518 (78.4%)	23 (67.6%)	
>3	143 (21.6%)	11 (32.4%)	0.143
Stage			
I	388 (58.7%)	19 (55.9%)	
II–IV	273 (41.3%)	15 (44.1%)	0.859
Differentiation			
Well/Moderate	522 (79.0%)	22 (64.7%)	
Poor	139 (21.0%)	12 (35.3%)	0.056

Classic activating mutation: EGFR exon 19 deletions and L858R point mutation; SD: standard deviation.

**Table 3:** Different EGFR mutations and response to EGFR TKIs.

Case	Mutation	Mutated exons	TKIs	Line	Response	RFS# (months)	OS# (months)
1	G719S+L861Q	18, 21	Gefitinib	1	PR	65.0	68.0+
6	V689L+L858R	18, 21	Erlotinib	3	PR	20.7	29.4+
18	E709K+G719C	18	Gefitinib	2	PR	14.8+	14.8+
21	G724S+S768I	18, 20	Erlotinib	2	SD	24.2+	24.2+
23	E709K+L858R	18, 21	Gefitinib	1	PR	37.5+	37.5+

^#^RFS and OS were calculated since taking TKIs.

TKIs, tyrosine kinase inhibitors; RFS, recurrence-free survival; OS, overall survival; PR, partial response; SD, stable disease.
