# Supplementary material for: EGFR Exon 18 Mutations in East Asian Patients with Lung Adenocarcinomas: A Comprehensive Investigation of Prevalence, Clinicopathologic Characteristics and Prognosis
Source: Sci Rep. 2015 Sep 10;5:13959. doi: 10.1038/srep13959 (PMC4564802; doi:10.1038/srep13959)
Supplement: Supplementary files [file srep13959-s1.doc]

**EGFR Exon 18 Mutations in East Asian Patients with Lung Adenocarcinomas: A Comprehensive Investigation of Prevalence, Clinicopathologic Characteristics and Prognosis**

Chao Cheng^1,2,+^; Rui Wang^1,2,+^; Yuan Li^2,3,+^; Yunjian Pan^1,2^; Yang Zhang^1,2^; Hang Li^1,2^; Difan Zheng^1,2^; Shanbo Zheng^1,2^; Xuxia Shen^2,3^; Yihua Sun^1,2^,*; Haiquan Chen^1,2,4,5^,*

**Authors’ Affiliations**:

^1^ Department of Thoracic Surgery, Fudan University Shanghai Cancer Center, Shanghai, 200032, China;

^2^ Department of Oncology, Shanghai Medical College, Fudan University, Shanghai, 200032, China;

^3^ Department of Pathology, Fudan University Shanghai Cancer Center, Shanghai, 200032, China;

^4^ Shanghai Chest Hospital, Shanghai Jiao Tong University, Shanghai, 200030, China;

^5^ Institutes of Biomedical Sciences, Fudan University, 130 Dong-An Road, Shanghai

200032, China.

^+^These authors contributed to this work equally and should be considered co-first authors.

***Correspondence** should be addressed to Haiquan Chen (E-mail:hqchen1@yahoo.com) or Yihua Sun (E-mail:sun_yihua76@hotmail.com)

**Address for correspondence**: Department of Thoracic Surgery, Fudan University Shanghai Cancer Center, 270 Dong’An Road, Shanghai 200032, China.

**Telephone**: +86-21-64175590

**Fax Number**: +86-21-62686511

**Supplementary Table S1.** Significant predictors of RFS and OS in univariate analysis of the 33 EGFR exon 18 mutations or classic mutations patients.

| **Variable** | **Category** | ***P*** |
| --- | --- | --- |
| RFS |  |  |
| Gender | Man vs. Female | 0.003 |
| Smoking history | Ever vs. Never | < 0.001 |
| Tumor size (cm) | ≤3 vs. >3 | < 0.001 |
| Stage | I vs. II-IV | < 0.001 |
| Differentiation | Well/Moderate vs. Poor | < 0.001 |
| OS |  |  |
| Smoking history | Ever vs. Never | 0.017 |
| Tumor size (cm) | ≤3 vs. >3 | < 0.001 |
| Stage | I vs. II-IV | < 0.001 |
| Differentiation | Well/Moderate vs. Poor | < 0.001 |
| Abbreviations: RFS, recurrence-free survival; OS, overall survival. | | |

**Supplementary Table S2.** Multivariate analysis of predictors of RFS and OS on 33 EGFR exon 18 mutations or classic mutations patients.

|  | **RFS** | | **OS** | |
| --- | --- | --- | --- | --- |
|  | ***P*** | **Hazard ratio (95%** | ***P*** | **Hazard ratio (95%** |
|  |  | **CI)** |  | **CI)** |
| Mutation |  |  |  |  |
| Single exon 18 | 0.646 | 1.173 (0.594-2.313) | 0.049 | 2.239 (1.005-4.989) |
| Complex exon | 0.906 | 0.958 (0.470-1.955) | 0.957 | N/A |
| 18 |  |  |  |  |
| Gender | 0.094 | 1.428 (0.941-2.166) | 0.322 | 1.373 (0.733-2.574) |
| Age | 0.830 | 1.001 (0.988-1.015) | 0.879 | 0.998 (0.978-1.019) |
| Smoking history | 0.001 | 2.184 (1.393-3.422) | 0.060 | 1.926 (0.973-3.810) |
| Tumor size | 0.019 | 1.397 (1.057-1.845) | 0.003 | 1.917 (1.247-2.947) |
| Stage | <0.001 | 4.763 (3.485-6.510) | <0.001 | 5.644 (3.064-10.396) |
| Differentiation | 0.002 | 1.608 (1.199-2.156) | 0.002 | 2.036 (1.310-3.165) |

Abbreviations: RFS, recurrence-free survival; OS, overall survival; N/A, not applicable.

| **Supplementary Table 3**. Primers and annealing temperatures for PCR and sequencing analysis. | | | | | |
| --- | --- | --- | --- | --- | --- |
|  | Gene | Exon | Primer sequence(5'-3') | | Annealing |
|  |  |  |  |  | temperature(°C) |
|  | *EGFR^a^* | E18 | F: | AGCATGGTGAGGGCTGAGGTGAC | 64 |
|  |  |  | R: | ATATACAGCTTGCAAGGACTCTGG |  |
|  |  | E19 | F: | CAGATCACTGGGCAGCATGTGGCAC | 64 |
|  |  |  | R: | AGGGTCTAGAGCAGAGCAGCTGC |  |
|  |  | E20 | F: | GATCGCATTCATGCGTCTTCACC | 64 |
|  |  |  | R: | TTGCTATCCCAGGAGCGCAGACC |  |
|  |  | E21 | F: | TAACGTTCGCCAGCCATAAG | 60 |
|  |  |  | R: | CGAGCTCACCCAGAATGTC |  |
|  | *EGFR^b^* | E18-21 | F: | TGAAGGCTGTCCAACGAATG | 61.5 |
|  |  |  | R: | AGGCGTTCTCCTTTCTCCAG |  |
|  | *^a^*primer for DNA based analysis; *^b^*primer for cDNA based analysis. | | | | |
